# Hsp70 and Calcitonin Receptor Protein in Extracellular Vesicles from Glioblastoma Multiforme: Biomarkers with Putative Roles in Carcinogenesis and Potential for Differentiating Tumor Types

**DOI:** 10.3390/ijms25063415

**Published:** 2024-03-18

**Authors:** Giusi Alberti, Christian M. Sánchez-López, Antonio Marcilla, Rosario Barone, Celeste Caruso Bavisotto, Francesca Graziano, Everly Conway de Macario, Alberto J. L. Macario, Fabio Bucchieri, Francesco Cappello, Claudia Campanella, Francesca Rappa

**Affiliations:** 1 Department of Biomedicine, Neurosciences and Advanced Diagnostics (BiND), University of Palermo, 90127 Palermo, Italy; giusi.alberti@unipa.it (G.A.); rosario.barone@unipa.it (R.B.); celeste.carusobavisotto@unipa.it (C.C.B.); fabio.bucchieri@unipa.it (F.B.); francesco.cappello@unipa.it (F.C.); claudia.campanella@unipa.it (C.C.); 2 Área de Parasitología, Departamento Farmacia y Tecnología Farmacéutica y Parasitología, Universitat de València, 46100 Burjassot, Spain; christian.sanchez@uv.es (C.M.S.-L.); antonio.marcilla@uv.es (A.M.); 3 Joint Unit of Endocrinology, Nutrition and Clinical Dietetics, Instituto de Investigación Sanitaria-La Fe, 46026 Valencia, Spain; 4 Euro-Mediterranean Institute of Science and Technology (IEMEST), 90139 Palermo, Italy; econwaydemacario@som.umaryland.edu (E.C.d.M.); ajlmacario@som.umaryland.edu (A.J.L.M.); 5 Department of Neurosurgery, Highly Specialized Hospital of National Importance “Garibaldi”, 95122 Catania, Italy; francesca.graziano@unict.it; 6 Department of Microbiology and Immunology, School of Medicine, University of Maryland at Baltimore-Institute of Marine and Environmental Technology (IMET), Baltimore, MD 21202, USA; 7 The Institute of Translational Pharmacology, National Research Council of Italy (CNR), 90146 Palermo, Italy

**Keywords:** glioblastoma multiforme, chaperone system, Hsp70, CTR, calcitonin receptor protein, EV, extracellular vesicles, liquid biopsy, biomarkers, early diagnosis

## Abstract

Glioblastoma multiforme (GBM) is a malignancy of bad prognosis, and advances in early detection and treatment are needed. GBM is heterogenous, with varieties differing in malignancy within a tumor of a patient and between patients. Means are needed to distinguish these GMB forms, so that specific strategies can be deployed for patient management. We study the participation of the chaperone system (CS) in carcinogenesis. The CS is dynamic, with its members moving around the body in extracellular vesicles (EVs) and interacting with components of other physiological systems in health and disease, including GBM. Here, we describe the finding of high amounts of Hsp70 (HSPA1A) and the calcitonin receptor protein (CTR) in EVs in patients with GBM. We present a standardized protocol for collecting, purifying, and characterizing EVs carrying Hsp70 and CTR in plasma-derived EVs from patients with GBM. EVs from GBM patients were obtained just before tumor ablative surgery (T0) and 7 days afterwards (T1); Hsp70 was highly elevated at T0 and less so at T1, and CTR was greatly increased at T0 and reduced to below normal values at T1. Our results encourage further research to assess Hsp70 and CTR as biomarkers for differentiating tumor forms and to determine their roles in GBM carcinogenesis.

## 1. Introduction

Glioblastoma multiforme (GBM) is the deadliest form of brain malignancy for which there is no effective therapy [1]. Currently, the standard of care for patients with GBMs is maximal surgical resection combined with chemo- and radiotherapy with the alkylating agent temozolomide (TMZ); however, median survival remains approximately 15 months after diagnosis (WHO malignancy grade IV) [2,3]. The failure of the current protocols is mostly the consequence of the intratumoral heterogeneity in each patient and the intertumoral diversity between patients, caused by the coexistence of multiple subclones within the same tumor [4]. GBMs are complex tumors characterized by a highly heterogeneous environment in which tumor cells rapidly adapt to the surrounding microenvironment consisting of neuronal, immune, stromal, and vascular cells, which in turn actively participate in the progression of GBM and therapeutical resistance [5]. Because of that heterogeneity, various biomarkers have been proposed for diagnosing glioblastoma at the early stages or for monitoring patient responses to treatment [6,7,8,9]. However, it is still necessary to improve the capabilities of differential diagnosis with new biomarkers and, most importantly, it is cogent to develop new and more efficacious treatment agents and protocols for GBM.

Over the last few decades, the role of extracellular vesicles (EVs) in cancer development and progression have been extensively investigated [10,11,12]. Efforts have been made to elucidate the EV cargo in relation to the source and to assess the practical and clinical value of EVs for diagnosis and patient monitoring. EVs are membrane-surrounded nanoparticles containing complex cargos, including proteins, lipids, and nucleic acids, which may become a part of the EV membrane, and some of them represent “molecular signatures” [13]. EVs are found in all biological fluids (e.g., blood, urine, and cerebrospinal fluid), and their compositions reproduce those of the cells in which they originate, thus providing a cellular sample that is relatively easily accessible for diagnostic purposes and for monitoring responses to treatment in cancer [11,12,13,14,15]. Research to date has shown that EVs in circulation in tumor patients are useful and offer practical advantages, because they are stable and accessible via minimally invasive procedures, allowing a regular follow-up over time to monitor tumor progression and therapeutic results [16]. EVs obtained from the liquid biopsy of tumor patients with no previous cancer history constitute a promising tool for early cancer diagnosis and for evaluating cancer progression [12,17].

GBM-derived EVs have been shown to stimulate angiogenesis, tumor cell migration, and glioma cell proliferation, as well as the evasion of apoptosis and the mounting of resistance to drugs [18,19,20]. GBM-derived EVs favor tumor invasiveness, a common feature of gliomas accounting for their very high local tumor-recurrence rates and consequent lethality [21,22]. The profile of EVs is usually characterized by the presence of CD9, CD63, and CD81 tetraspanins (i.e., biomarkers ubiquitously present on EVs from most cell types), and these markers may change their quantitative levels, reflecting pathological conditions [23]. Elevated CD81 was found to strongly correlate with a decreased overall survival of GBM patients [23]. Alix and TSG101 are proteins involved in the formation of multivesicular bodies (MVBs) and, since they are present in the GBM-derived EVs, they are also used as markers [24]. GBM-derived EVs are enriched in immunostimulatory molecules (MHC I/II), cytoskeleton molecules (actin, myosin, and tubulin), membrane-trafficking proteins (Rab GTPases), and heat shock proteins (Hsps), for instance Hsp60, Hsp70, and Hsp90 [25,26]. These Hsps are molecular chaperones whose expression is upregulated following stressful stimuli, such as heat and physical exercise, as well as in pathological conditions [27,28,29,30,31,32]. We recently studied the tissue levels of Hsp27, Hsp60, Hsp70, and Hsp90 in GBM samples and GBM cell lines, and observed a downregulation of Hsp70 compared to other Hsps [33]. A cornerstone of GBM is the calcitonin receptor (CT receptor, CTR), found in 76–86% of patient biopsies [34]. The calcitonin receptor family includes the CTR and the calcitonin receptor-like receptor (CLR). The CTR can form complexes with RAMP (receptor activity-modifying protein) [35]. These proteins can modify the activity of the receptor to generate receptor phenotypes with different binding specificities for the calcitonin (CT) peptide family [36]. The CT receptor belongs to subclass B of the superfamily of the seven-transmembrane domain G protein-coupled receptors (GPCRs) [37,38,39]. Under physiological conditions, CTR expression in the human brain has been revealed in the hypothalamus, limbic system, and circumventricular organs of the brain stem, but not elsewhere [40]. In patients with GBM, CTR is found almost exclusively in the cortex of frontal and temporal lobes, where it is not normally expressed [41,42]. Furthermore, the CTR is subject to alternative splicing, which includes 16 amino acids in the intracellular loop 1 (ICL1) of the receptor, resulting in the formation of two conventionally defined variants: the CRT-negative variant, or CTR_a_, and the CRT-positive variant, or CTR_b_ [34].

To further characterize the molecular phenotype of GBM patients and search for patterns that might help in early diagnosis, we analyzed the presence of Hsp70 and CTR in EVs isolated from the plasma of GBM patients compared to those obtained from healthy individuals. We now propose that these two molecules are promising diagnostic biomarkers profiting from the dynamic window offered by EVs, and provide a standardized protocol for use in diagnosis and in elucidating the role of the chaperone system (CS) and CTR in GBM carcinogenesis.

## 2. Results

### 2.1. Plasma-Derived EVs Show Differences in Their Size and Number between GBM Patients and Healthy Controls

**Figure 1:**
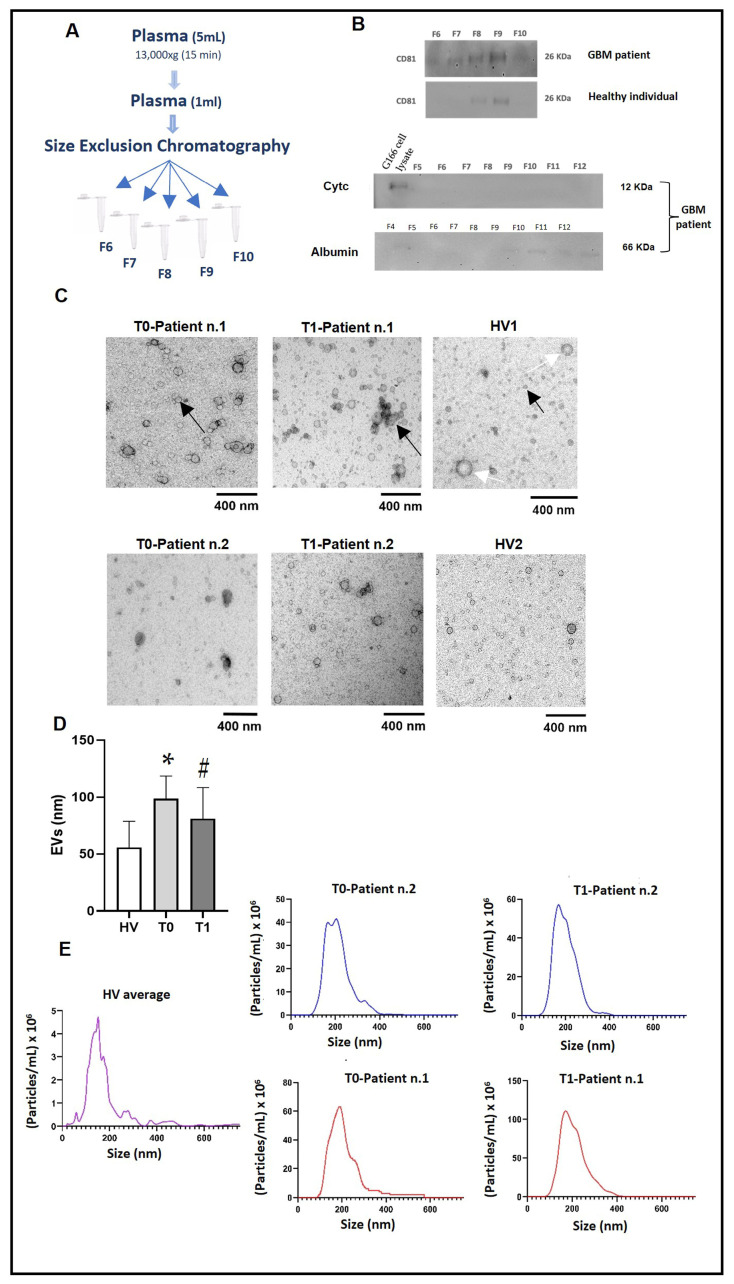
Evaluation of the fractions of EVs isolated by SEC from the plasma of GBM patients and HVs (healthy volunteers). (**A**) Diagram representing the workflow of EV separation by SEC. (**B**) Lysates of EVs from fractions f5–f12 were separated by SDS-PAGE and analyzed by immunoblot for a specific EV marker, namely CD81 (loading 50 μg). The top line refers to fractions f6–f10 from a patient with GBM, while the line immediately below refers to fractions f6–f10 from a healthy donor. Cytochrome C (Cyt C), which was present only in the total cell lysate (TCL) of a glioblastoma staminal cell line (G166), was used as a negative control. Albumin was chosen to evaluate the amounts of impurities in isolated EVs. (**C**) Representative transmission electron microscopy (TEM) images of EVs (fraction f9) derived from two GBM patients, at T0 (EVs isolated before surgery) and T1 (EVs isolated after surgery), and HV1 and HV2 (healthy volunteers) (black arrow: EVs; blank arrow: contamination). Scale bar: 400 nm. (**D**) Statistical analysis of the average size of EVs present in 9 fractions of all pathological and healthy samples after the observation of TEM images using ImageJ software version 1.53k (nm: unit of measurement used is nanometers); *: increased significantly from the HVs (*p* < 0.0001); #: increased significantly from the HVs (*p* < 0.03). (**E**) Representative image of the determination of the number of particles and the dimensions of the EVs present in the f9 of two patients compared to the EVs isolated from HV1 and HV2 (HV average) by NTA. A high amount of EVs was readily detected in T0 and T1 samples from patients, and less so in EVs derived from HVs. Four captures of 60 s each were recorded.

EVs were isolated from plasma obtained from patients and healthy individuals by differential centrifugation, followed by SEC (size exclusion chromatography) (Figure 1A). Upon analysis, twelve fractions were found to contain EVs. Each fraction was assessed by Western blot, TEM, and NTA, showing that EVs mainly eluted in fractions 7–9, while plasma proteins eluted in later fractions (Figure 1B). Western blot demonstrated that the fractions 7–9 were enriched in EVs, as determined by the presence of the transmembrane tetraspanin CD81 (a reliable EV marker [13]), whereas the most abundant free proteins in plasma were eluted in later fractions (f10–f12), such as human serum albumin (HSA) (Figure 1B). Fractions f7–f9 contained a typical marker of EVs, but were virtually free of mitochondrial proteins (cytochrome C as negative marker, Cyt C) (Figure 1B). We evaluated the presence of albumin as a non-EV structure that often co-isolated with EVs isolated by SEC, as recently reported [43,44]. These results show that SEC is an efficient method to isolate EVs from biofluids without significant amounts of plasma protein-bound impurities.

Subsequently, TEM was used to investigate the structure and dimensions of the EVs in fractions f7–f9 for each sample. Figure 1C shows TEM images of the EVs present in the f9 of two patients with GBM, compared to two healthy donors (HV1 and HV2). EVs with typical structures were detected in the EVs isolated before surgery (T0) and in the EVs isolated after surgery (T1), while in the healthy volunteer (HV) samples, poorly defined round structures were observed, suggesting the presence of contaminants (e.g., lipoprotein-like structures) (Figure 1C). The analyses of TEM images showed a significant increase in the average size of EVs isolated from GBM patients before (T0) and one week after the surgery (T1) in comparison with those isolated from healthy controls (Figure 1D), as previously reported in the literature [45].

By using NTA, we analyzed the number of particles and the size profile of EVs in the fractions of interest (f7–f9) for each patient and healthy volunteer (HV). In these fractions, we found the highest concentration of particles (Figure 1E). The number of particles detected by NTA was 50 ± 80 × 10^8^ particles/mL, with a mean size of 200 nm at T0 and T1 in GBM samples, whilst the amount of EVs was lower in healthy controls: 25 ± 40 × 10^8^ particles/mL with a mean size of 100–150 nm (Figure 1E). The size profile detected by TEM (Figure 1C) and the number of particles detected by NTA (Figure 1E) were similar in T0 and T1, with a characteristic peak around 200 nm. However, there were statistically significant differences in the EV concentration and average size between the GBM patients and the healthy controls (Figure 1C–E).

### 2.2. Analysis of Pooled EV Samples Showed That EVs Isolated from GBM Patients Were Significantly More Enriched in Protein Than EVs Obtained from HVs

**Figure 2:**
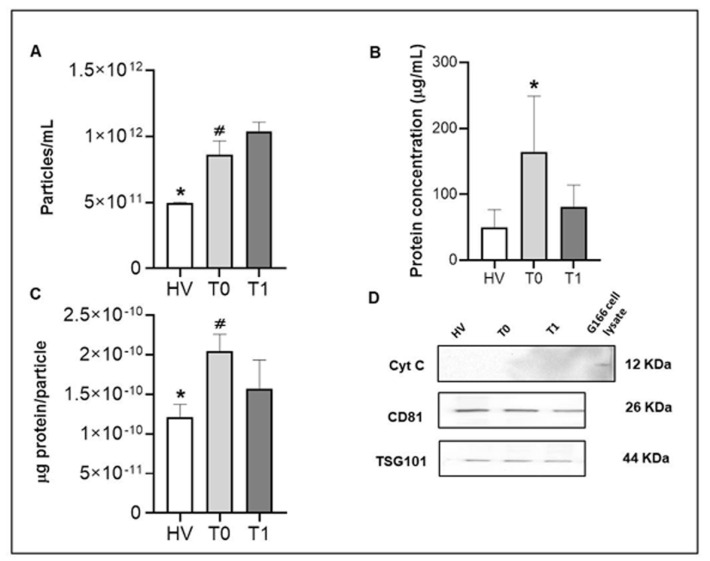
Determination of the enrichment in EVs and the protein cargo into EVs for pathological and healthy samples. (**A**) Determination of the number of particles (EVs) in the pools of EV-enriched fractions of patients with GBM, at T0 and T1, and HVs; *: decreased significantly from T0 and T1 (*p* < 0.0001); #: decreased significantly from T1 (*p* < 0.0001). (**B**) Determination of the protein content of the pools of SEC EV-enriched fractions of patients with GBM, at T0 and T1, compared to HVs. Our results show that the amounts of proteins differed significantly between T0 and T1 samples, with higher protein concentrations in T0 with respect to T1 and HV; *: increased significantly from HV and T1 (*p* < 0.0001). The pool of EV-enriched fractions of T0 provided a relatively higher protein yield than the samples from T1 patients and HV. (**C**) The ratio between protein concentration and the number of particles; *: decreased significantly from T0 and T1 (*p* < 0.0001); #: increased significantly from T1 (*p* < 0.0001). (**D**) Equal loading of protein lysates of pools of EV-enriched fractions from GBM patients and healthy donors were separated by SDS-PAGE and analyzed by immunoblot for CD81 and TSG101 as positive controls, and Cyt C as a negative control. Images are representative of at least 3 independent experiments.

The results reported above indicate that most of the EVs were contained in fractions f7–f9, and thus they were pooled and concentrated for further studies. We determined the particles’ number and the protein concentration in the pooled samples by NTA and Bradford tests, respectively. The particle concentrations observed at T0 and T1 in patients with GBM were different, with a statistically significant increase at T1 compared to T0 (*p* < 0.0001), as illustrated by the results shown in Figure 2A.

Higher significantly numbers of EVs were found in T0 and in T1 compared to the controls from healthy donors (*p* < 0.0001) (Figure 2A). Once this was resolved, we moved on to determining the protein concentration of the pooled fractions. A significant increase in total protein content was detected in the pooled fractions from patient samples before surgery (T0) relative to T1 and HVs samples (*p* < 0.0001) (Figure 2B). Accordingly, the determination of the ratio between the protein concentration and the particle number for all samples and healthy subjects showed that EVs isolated at T0 (2.05 ± 1.25 × 10^−10^ μg of protein/particle) were more enriched in protein than EVs obtained at T1 (1.55 ± 1.05 × 10^−10^ μg of protein/particle) (*p* < 0.0001), and than HVs (1.08 ± 2.55 × 10^−11^) (*p* < 0.0001) (Figure 2C). The results corroborated that EVs isolated before surgery (T0) were enriched in protein and that EVs isolated before surgery (T0) were purer than those isolated after surgery (T1), and even more so when compared with EVs isolated from healthy individuals.

Positive and negative markers of EVs were also analyzed by Western blot. CD81 and TSG101 markers showed similar levels in the EVs of all samples, whereas no Cyt C was detected (Figure 2D). These results demonstrate that the EVs collected using SEC methods were enriched in EV fractions, as defined by MISEV criteria [13].

### 2.3. Mass Spectrometry of EVs Produced by GBM Patients and Healthy Controls

**Figure 3:**
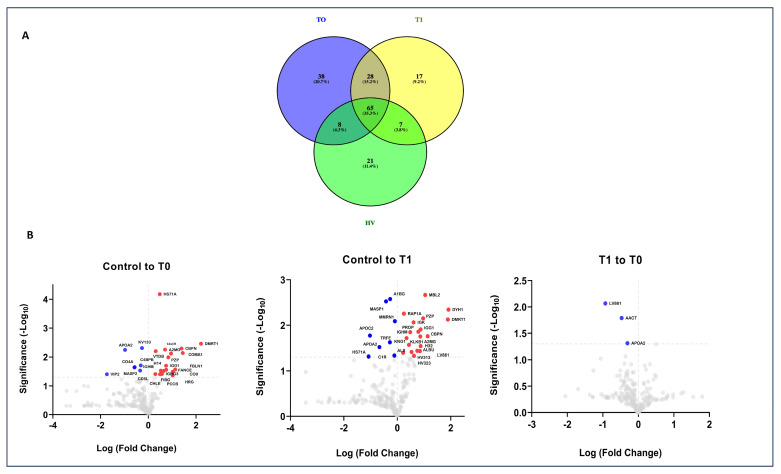
Proteomes of EVs from T0, T1, and HVs. (**A**) The Venn diagram represents the number of shared and unique proteins of the three groups. (**B**) The Volcano plot shows comparisons between the control and T0, control and T1, and T1 and T0; the red and blue circles represent *p* < 0.05 and Log2 fold change, respectively; the gray circles have no statistical significance.

Label-free LC-MS/MS analysis was performed to identify proteins in EVs and carry out comparisons between the cancer and healthy groups. A total of 202 proteins were present in isolated EVs (detailed information shown in Table S1). There were 139 and 117 proteins in isolated EVs at T0 and T1, respectively, and 101 in HV samples (Table S1 and Figure 3A).

**Figure 4:**
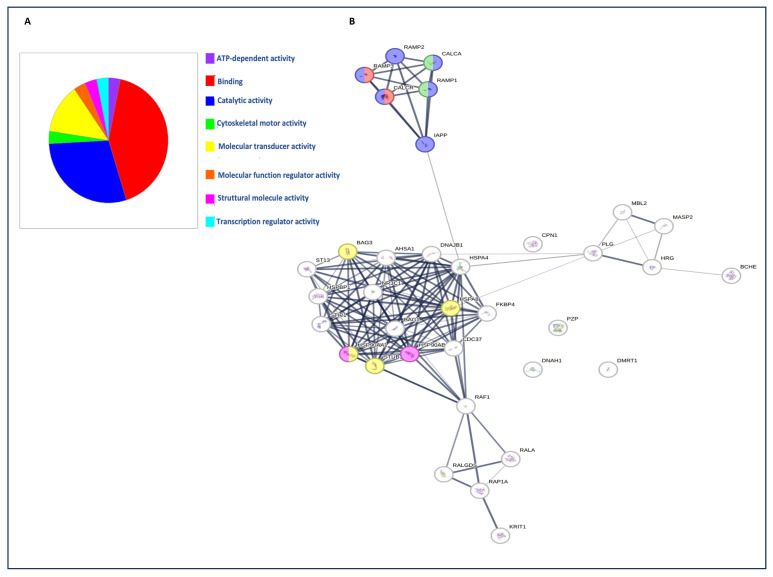
Proteomic analysis of differentially expressed proteins. (**A**) The pie chart shows the molecular functions of the identified proteins according to gene ontology (GO). (**B**) Protein–protein interaction analysis was performed using STRING software version 11.0, with the edges indicating both functional and physical protein associations; the line thickness indicates the strength of the data support.

The overlap of proteins between groups was observed in a Venn diagram to evaluate the reproducibility of the proteins discovered among the groups (Figure 3A). A total of 65 proteins were shared by the control and GBM groups, and 38 (20.7%) and 17 (9.2%) proteins were found only in the T0 and T1 groups (Table S1, Figure 3A). A label-free differential expression analysis was carried out to identify the proteins that showed the largest quantitative differences between samples. A *p*-value < 0.05 was adopted as cutoff limit, and the list of the differentially abundant proteins is shown in Figure 3B and Table S2. By applying a log fold change >2 cutoff, the results showed that 17 proteins were increased in EVs obtained from GBM patients at T0 compared to the healthy controls, and 19 proteins were significantly changed in the EVs derived from GBM patients at T1 when compared to the healthy groups, while the levels of only 3 proteins were different between T1 and T0 (Figure 3B and Table S2). The data show that 202 proteins were identified in this study, of which 39 were elevated in quantity, and 163 were decreased (Figure 3B and Table S2). The top five proteins that showed a significant quantitative difference between the EVs derived from the GBM groups and the control were CHLE, CALCR, CBPN, HRG, and HS7A1 at T0, and MBL2, DYH1, RAP1A, PZP, and DMRT1 at T1 (Figure 3B and Table S2). Functional enrichment analyses of differentially abundant proteins in EVs were performed to determine if there were functional differences between the proteins at T0 and T1, using a gene ontology (GO) analysis (using the PANTHER-GO system, version 18.0; https://pantherdb.org/chart, accessed on 1 March 2024) (Figure 4A).

A molecular function study revealed that most of the differentially abundant proteins between EVs isolated from GBM patients and healthy controls were involved in catalytic and binding activity (Figure 4A). Protein–protein interactions between the differentially abundant proteins identified in Evs isolated from GBM patients were also investigated using the STRING database for functional protein association networks (https://string-db.org, version 11.0) (Figure 4B). Finally, we compared the proteins quantified in GBM Evs with those included in the ExoCarta (http://www.exocarta.org/) and Vesiclepedia (http://www.microvesicles.org/) databases and observed that the overlap rates of our EVs’ proteome were 78 and 84%, respectively (Table S1).

### 2.4. Differences in Cargo of EVs Isolated from GBM Patients and Healthy Subjects Reveal the Presence of Cancer-Related Markers

**Figure 5:**
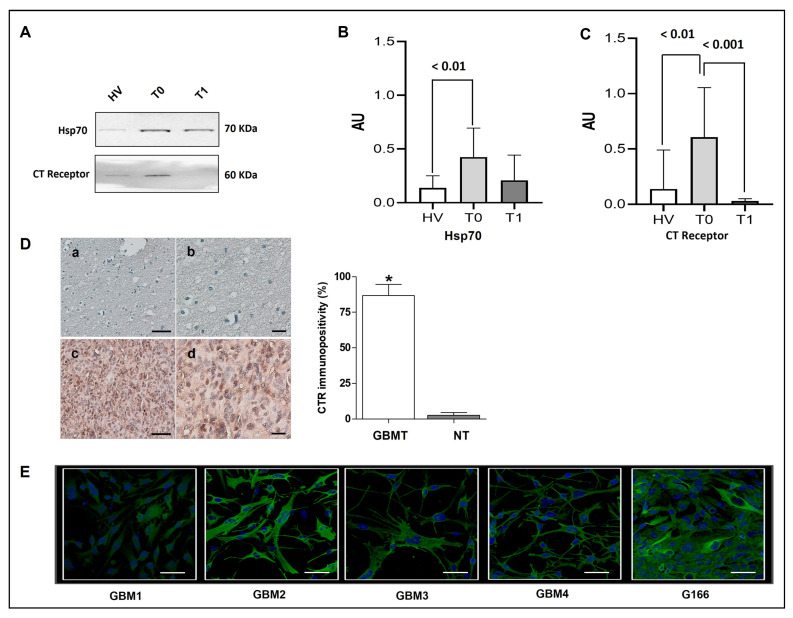
Hsp70 and CTR are present in SEC-purified EVs from GBM patients. (**A**,**B**) Western blot and statistical analysis for Hsp70, and (**A**,**C**) for CTR (loading 50 μg, and we used Cyt C as a negative control). From the left: lane 1, EVs derived from the plasma of healthy individuals (HV); lane 2, EV lysates derived from the plasma of GBM patients at T0; lane 3, EV lysates derived from the plasma of GBM patients at T1. The images are representative of at least 3 independent experiments. (**D**) Representative images of immunohistochemistry for CTR in normal tissue (NT) (**a**,**b**), and GBM tissue (GBMT) (**c**,**d**). (**a**,**c**): Magnification, 200×; scale bar, 50 µm. (**b**,**d**): Magnification, 400×; scale bar, 20 µm. The histogram shows the statistical analysis of the IHC data (* *p* ≤ 0.01). (**E**) Immunofluorescence images of CTR showing its presence in the primary and secondary cell lines. A specific primary antibody for the receptor and a secondary antibody conjugated with FITC (green fluorescence) were used, and the cell nuclei (blue) were stained with DAPI. Magnification, 400×.

To evaluate differences in the cargo of EVs isolated from GBM patients and HVs, Western blot was performed for two proteins of interest, Hsp70 and CTR. Hsp70 was mainly detected in T0 and T1 samples, whereas only a light band was visible in Western blots of HV samples (Figure 5A). This result showed significantly higher amounts of Hsp70 (HSPA1A) in EVs isolated from GBM plasma before surgery than in the EVs isolated from HVs (*p* < 0.01) (Figure 5A,B).

In an attempt to identify potential cancer markers in EVs, we investigated the levels of CTR in our samples. The analysis revealed that EVs from T0 samples contained higher levels of the CTR protein compared to EVs isolated from HV and T1 (*p* < 0.01 and *p* < 0.001, respectively) (Figure 5A,C). The results showed a tendency towards lower levels of Hsp70 (HSPA1A) in EVs isolated after surgery (T1) compared to the levels before surgery (T0), while the lowest levels were observed in EVs obtained from healthy subjects. For CTR, the results showed a significant enrichment of EVs isolated from GBM patients at T0 compared to EVs obtained from GBM patients at T1 and HVs.

To determine the localization and the tissue levels of CTR, we performed an immunomorphological analysis with immunohistochemical methods on GBM tissue sections from all patients and normal tissue sections. As shown in Figure 5D, a high immunopositivity, namely 85 ± 7.8% of the cells, was found in the GBM tissue (GBMT) in contrast to 3 ± 1.9% of the cells in normal tissue (NT). In the GBM samples, positive staining was observed in the cytoplasm, with a diffuse pattern in all cells, as well as in the nucleus. In the control group (NT), however, low staining was observed only in the cytoplasm. The statistical analysis showed a significant difference (*p* ≤ 0.01) between the two groups as shown in the histogram (Figure 5D). The CTR protein was also assessed by immunofluorescence in cell lines already employed in a previous study, specifically four primary cell lines and a secondary GBM cell line, i.e., G166 [33]. As previously reported, the primary GBM cell lines were from resected tissue obtained during surgical procedures, while G166 is a secondary GBM stem cell line [33]. In our study, the CTR was detected to be mainly localized in the cytoplasm (perinuclear domain), as well as in the primary and secondary cell lines (Figure 5E). These data confirm that the GBM tissue and derived cell lines contained high levels of the CTR protein, which is bound to be carried by EVs. Future studies will address this possibility.

## 3. Discussion

Progress has been made in the understanding of the pathogenesis and manifestations of GBM, yet patients still face poor overall survival and limited treatment options. Currently, the diagnosis of GBM is based on imaging techniques combined with histological studies on tissue biopsies to identify molecular biomarkers, such as IDH, TP53, and EGFR [46]. Over the past decade, clinical oncology research has developed a new frontier area with an emphasis on the analysis of biological fluids such as blood, e.g., using what has been named liquid biopsy, which offers the possibility of repeated sampling throughout treatment in a minimally invasive manner. A liquid biopsy can sometimes reveal information about the tumor even before clear clinical manifestations appear [47]. Biological fluids are enriched in various biomarkers, part of which are carried by EVs, and these are very convenient from the practical viewpoint because of their stability in the circulation and the characteristics of their cargo [48,49,50]. The identification of specific distinctive molecular signatures in EVs released by cancerous cells is encouraging clinical applications of liquid biopsy targeting EVs [51]. Currently, the www.ClinicalTrials.gov database lists three preclinical studies highlighting their potential for cell-free therapy in clinical practice, accessed on 1 September 2023. One of these (NCT04993378) evaluates the potential of four plasma EV-derived proteins in immunotherapy and the monitoring of disease progressions in gastric cancer patients, while (NCT05798338) screens potential markers in the circulating EVs from patients with breast cancer at specific stages. However, this goal remains challenging, since no validated markers specific to GBM and their variations along the course of the disease are yet available.

We previously reported the absence of Hsp70 in GBM tissue as well as in derived primary and secondary cell lines [33], and those results led us to investigate the possibility of detecting Hsp70 outside the tumor cells, for example in EVs. Hsp70 can be engulfed within EVs, which gain the circulation, and thus are able to release the chaperone at critical points near to and far from the tumoral mass. This transportation could be a way to increase tumorigenicity, although there is also information that indicates that Hsp70-enriched EVs possess negative immunomodulatory activities on tumor growth [52,53,54]. Notably, EVs act not only at short distances between neighboring cells within the tumor mass, but exert long-distance effects eliciting potentially higher pathogenicity [12,55,56].

It is known that Hsp70 contributes to an aggressive tumor phenotype and resistance to therapy, and it is an indicator of poor prognosis [57,58,59]. Hsp70 expression is induced by various types of stress and is cytoprotective by interacting with different molecules involved in the cell-death pathway [60,61].

In the present study, we isolated EVs from the plasma of GBM patients and HVs and characterized them for the size, shape, and presence of the typical EV markers CD81 and TSG101 (Figure 1B–D and Figure 2D). Specifically, TEM images showed a population of round-shaped structures with a size distribution in good accordance with NTA measurements. (Figure 1C–E). We observed the presence of vesicles with dimensions between 100 and 200 nm or slightly larger and the typical shaped morphology of EVs (Figure 1C) [62]. Furthermore, the SEC-isolation method yielded EV fractions with enriched protein content at T0 compared to T1 and healthy controls (Figure 2B,C). The characterization of EVs in the current study (Figure 1B and Figure 2D) displayed concordance with MISEV2018 guidelines when evaluating known markers of extracellular vesicles, such as tetraspanins and proteins involved in EV biogenesis [13]. We show that Hsp70 is secreted via EVs and, thus, could be considered as a candidate to follow, and determine whether it has a differential diagnostic potential or a carcinogenic role in GBM.

The proteomic profile of EVs derived from GBM patients and healthy subjects detected 202 proteins, of which 39 were present at levels that differed between the groups compared (Table S2, Figure 3A,B). This protein heterogeneity suggests that the content of EVs is variable between EVs isolated at T0, T1, and HVs, and that this difference in the EV cargo probably depends on the pathological and non-pathological condition. These 39 proteins identified belong to the categories of binding-related molecules, proteins with catalytic activity, and proteins with structural functions, as well as proteins involved in transcriptional regulation (Figure 4A). We speculate that the enrichment of these proteins is a result of protein overlap with the other main pathways (Figure 3B and Figure 4A). This indicates that binding-related components are privileged for cargo into EVs (Figure 4A). Among them, CHLE, CALCR, CBPN, HRG, and HS7A1 were more abundant in T0, and MBL2, DYH1, RAP1A, PZP, and DMRT1 were more abundant at T1 when compared to the healthy controls (Figure 3B). CHLE is an esterase with broad substrate specificity, which contributes to the inactivation of the neurotransmitter acetylcholine and the degradation of neurotoxic organophosphorus esters, which are mechanisms underlying the development of neurotransmission disorders in the brain or cancer [63]. CACLR is a calcitonin receptor, whose activity is mediated by G proteins that activate adenylate cyclase. It is present during the life cycle of organisms, both in physiological and pathological conditions, such as GBM [34,35]. CBPN is a plasma metalloprotease involved in vascular development, and its role in GBM is unclear [64]. HRG is involved in fibrinolysis and coagulation processes, and plays a role in inflammation and immunity, and its role has not been investigated in GBM [65]. MBL2 is a mannose-binding protein that plays a critical role in the immune response, and is capable of binding glioma cells in vitro [66]. DYH1, known as flagellar dynein, is involved in the transport of cellular cargo along cytoskeletal microtubules towards the cell center. Its role has not currently been studied in GBM [67]. RAP1A is a protein belonging to the Ras subgroup, which is widely studied for its contribution to the malignant progression of numerous human cancers, including GBM [68]. PZP is a protein belonging to the α-2-microglobulin superfamily, which is involved in inflammatory responses and immune cell activation in cancer [69]. DMRT1 is a transcriptional regulator that plays a key role in male sex determination and differentiation by controlling testicular development and male germ cell proliferation [70]. Because our work is directed toward the identification of biomarkers for the early diagnosis of GBM, we focused on CACLR, considering its cellular functions as a tumor suppressor, which are currently little known, and its ability to interact with various chaperone molecules, including some belonging to the Hsp70 family (Figure 4B). Furthermore, our proteomic analysis identified proteins involved in the canonical and alternative complement pathways, in the metabolic pathway, in inflammatory pathways, and more (additional information is presented in Table S3 [71,72,73,74,75,76,77,78,79,80,81,82,83,84,85,86,87,88,89,90,91,92,93,94,95,96,97,98,99]). The differentially expressed proteins were further analyzed using the STRING database to derive an interaction network and potential signaling pathways, which may reveal the tumorigenic mechanism underlying GBM (Figure 4B). Overall, our data suggest that EVs can be exploited for patient classification, patient follow-up, and recurrence monitoring.

Compared to healthy individuals, the patients with GBM had higher levels Hsp70 in their plasma EVs before surgery (T0). These data are in line with other studies reporting an enrichment in the Hsp70 of EVs isolated from cancer patients [52,57,100,101]. In this study, significantly higher Hsp70 levels were detected in EVs from GBM patients compared with healthy individuals, and the Hsp70 levels decreased after ablative surgery (Figure 5A,B). This finding is encouraging and presents Hsp70 (HSPA1A) as a prospective biomarker to follow in relation to specific features of GBM that might shed light on the participation of the chaperone system in the carcinogenic mechanism of GBM. Our data also suggest that it would be worth evaluating the diagnostic clinical value of Hsp70 levels in circulating EVs (liquid biopsy) in GBM patients, implementing longitudinal follow-up studies with many patients.

GBMs originate from the malignant transformation of astrocyte-glial precursors [102], and are characterized by a high cellular heterogeneity, which also includes a subpopulation of cells displaying stem cell characteristics (GSCs, glioma stem-like cells) that are involved in tumor progression [103,104,105]. Here, we discuss the differential diagnostic potential of CTR protein in EVs isolated from the plasma (liquid biopsy) of GBM patients. CTR is encoded in a gene located in chromosome 7q21.3, in a region of DNA that is frequently amplified in GBM tumors [38,106]. The *CALCR* gene is upregulated by the transcription factor Sp1 involved in the regulation of genes key for stress responses in various tissues, including GBMs [107]. Several studies reported that the expression of CTR is restricted in glioma cells [34,35,38,108]. Information available in the Human Protein Atlas (https://www.proteinatlas.org/CALCR/brain, accessed on 1 September 2023) and immunohistochemistry analyses indicate that the CTR expression occurs only in the hypothalamus, limbic system, and circumventricular organs in the brain stem in physiological conditions [40,109], but it is not expressed in the frontal and temporal lobe sites of GBM at the onset [41]. Furthermore, high levels of CALCRL mRNA were found in human glioblastoma cancer stem-like cells [110,111]. The *CACLR* gene undergoes alternative splicing that leads to an upregulation of the positive insert isoform (CTR_b_) with an unchanged total CALCR mRNA in GBM tissue; nonetheless, further studies are necessary to confirm this result [34]. In Western blotting experiments, the primary antibody used against CTR binds an intracellular epitope of the CTR, revealing a band that belongs to a protein of the expected molecular weight, and the band is consistent with the molecular segment predicted by the amino acid sequence, which corresponds to the predicted glycosylated protein (CTR_a_) confined to the intracellular domain [112,113].

**Figure 6:**
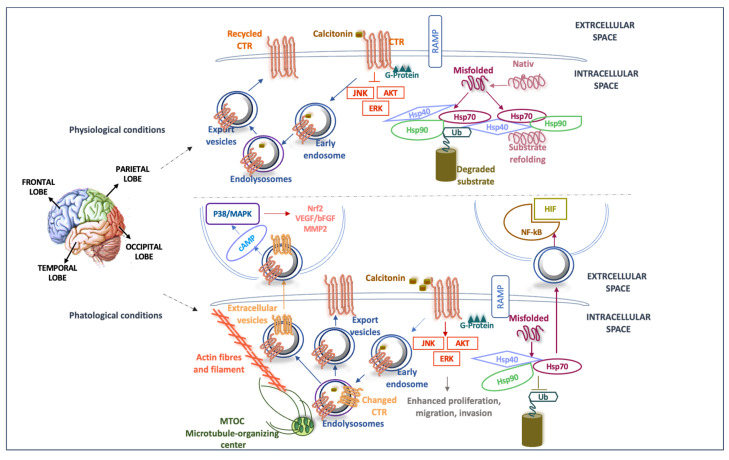
Scheme with hypothetical roles of Hsp70 and CTR in GBM pathogenesis. The figure includes the anatomical regions of the onset of GBM in the central nervous system and the pathways involving Hsp70 and CTR in physiological and pathological conditions, including their involvement in GBM. Because they are released by the tumor in EVs, they are potentials markers collectable by liquid biopsy, and thus are useful for early diagnosis and disease monitoring. As part of the physiological receptor cycle, the CTR is incorporated into the plasma membrane in which, following binding to its ligand, it undergoes a conformational change that leads to the regulation of various downstream signaling pathways (some of which are known to regulate various properties of cancer cells). Subsequently, the CTR is internalized in the cytosol within endosomal vesicles in which, with its carboxyl terminus facing outwards into the cytoplasm, it participates in trafficking within the cytoskeleton along microtubules [118], to be recycled onto the plasma membrane. Mutations in the gene coding for the CTR could, under conditions of cellular stress, inhibit the function of the protein, with the consequent deregulation of downstream signaling pathways. The possible alternative splicing of the CTR mRNA (often an associated oncogenesis process) would result in an increase in the expression of the shorter isoform of CTR (changed CTR in orange), which could be conveyed toward the extracellular space through EVs and, subsequently, would activate CTR signaling pathways in non-tumoral cells, or can be uptaken by GBM cells, increasing their proliferation, motility, invasiveness, and resistance to therapy. Under normal conditions, Hsp70, in cooperation with Hsp40 and other co-chaperones, assists in the folding of newly synthesized proteins, restores the native conformation of partially denatured proteins, and directs irreversibly damaged proteins to the ubiquitin–proteasome system or lysosomes for elimination. Under proteotoxic stress conditions, Hsp70 could be secreted into the extracellular space as a free protein or exported via EVs, depleting the intracellular space of the chaperone, and allowing the accumulation of misfolded proteins in the cytosol. In target cells, Hsp70 participates in many tumor-activating pathways [28,34,74].

The CTR isoforms present a different tissue distribution, as well as a different functional efficiency in the regulatory activity of downstream signal molecules. RT-PCR analysis demonstrated that CTR_b_ represents the isoform that is mainly localized in the central nervous system and is primarily present in membranous structures close to the nucleus, while CTR_a_ shows a broader distribution in vivo [34,111,112,113]. Importantly, the presence of the additional sequence in CTR_b_ leads to a decrease in or loss of functions related to the intracellular calcium mobilization and the binding of downstream molecules, thus being less efficient than CTR_a_ [34,112,113,114]. It has also been observed that mutations or polymorphisms in the *CALCR* gene could significantly decrease or abrogate the function of the protein with a consequent impact on the downstream signaling pathways (see Figure 6). For instance, it has been observed that somatic mutations in the *CALCR* gene led to a loss of the function of the protein in glioma cells, which correlated with a poor prognosis [110]. Instead, the polymorphism in the start codon of the CTR_a_ variant leads to the presence of a proline or a leucine, with a decrease in the maximum cellular response when the exome encodes leucine in the c-terminal tail of the protein (CTR_aLeu_) [115]. It was reported that CTR_a_ is involved in the activation of known CTR signaling pathways, including the ERK1/2 and p38 MAP kinases, and Ca^2+^ mobilization, which is typically altered in many cancers, including GBM (Figure 6) [116]. Of note, p38 MAPK is upregulated in GBM cell lines as well as in GBM patients [117].

In this work, we detected the glycosylated CTR isoform in EVs isolated from the plasma of patients with GBMs before surgery (T0), with an almost complete absence of the molecule after ablative surgery (T1), as well as in healthy individuals (Figure 5A,B), suggesting that the glycosylated protein could be a marker of the more aggressive GBMs with a poor response to treatment [80]. In order to investigate the CTR expression, we compared the variation in CTR expression levels between normal and tumor GBM tissues (Figure 5C). Compared to normal tissues, CTR was markedly elevated in the GBM tissue (Figure 5C). High levels of protein expression of CTR in primary and secondary GBM cell lines are shown in Figure 5D. In addition, a significant correlation between CTR levels and clinical variables, such as age, IDH wild-type or mutant, and poor prognosis, has been revealed, suggesting that CTR might stratify GBM tumors differently and allow the monitoring of the treatment response. In summary, we found significantly different levels of Hsp70 and the CTR protein in EVs isolated from GBM patients before surgery, and these levels significantly decreased after surgery. These results could allow a precise stratification of patients with more aggressive types of the disease, thus guiding the therapeutic decision. Furthermore, the significant decrease in the expression levels of the CTR protein after surgery could be used to monitor the risk of tumor recurrence via simple liquid biopsies. In addition, since Hsp70 and CTR also interact with several pathways involved in tumorigenesis, CTR could be considered a convenient therapeutic target in GBM treatment, as has been argued elsewhere [29,118] (Figure 6). Looking ahead, Hsp70 and CTR will be more fully characterized in in vitro and in vivo studies, in large cohorts of both GBM patients and healthy controls. This should: (a) increase our understanding of their pathogenic roles; (b) allow the evaluation of their usefulness as markers for early tumor detection and for monitoring tumor progression; and (c) provide clues to direct efforts toward the development of therapeutic agents specific to GBM types.

## 4. Materials and Methods

### 4.1. Patients and Blood Samples

Plasma was obtained after informed consent from GBM patients (n = 15) and healthy donors (n = 15). Detailed information on the clinical and molecular characteristics of the GBM patients enrolled in this study is shown in Table 1. This study was included in a scientific project approved by the Ethics Committee of the University Hospital AUOP Paolo Giaccone of Palermo (number 11/2018). At the time of the surgery (T0) and seven days after it (T1), 5 mL of blood were taken from patients with histologically confirmed GBM. Healthy controls were blood donors matched for age and sex, and were collected as morning samples relative to the timing of the plasma patient collections. For all samples, blood was collected in EDTA and centrifuged at 2500× *g* for 20 min prior to plasma storage at −80 °C.

### 4.2. Isolation of Extracellular Vesicles via Size Exclusion Chromatography

The isolation of EVs from plasma was performed as previously described [119]. Briefly, EVs were isolated from plasma using size exclusion chromatography (SEC) columns. We used 10 mL in-house packed columns using Sepharose CL-2B (Sigma-Aldrich, St. Louis, MO, USA), with a size exclusion limit of 75 nm. For the preparation of the columns, plastic tubes with a filter near the column outlet (SPE polypropylene tube—polyethylene FRIT) were used. The flow rate of the filtered phosphate buffered saline (PBS) effluent was controlled manually. Plasma samples stored at −80 °C were thawed overnight at 20 °C, and then centrifuged at 3000× *g* for 10 min at 4 °C with protease inhibitors (P8340 protease inhibitor cocktail) to remove precipitates, cell debris, and larger vesicles prior to loading on the column. Next, the plasma samples were centrifuged at 13,000× *g* for 15 min a 4 °C, and then filtered with 0.22 µm filters. Finally, the plasma samples were concentrated down to 1 mL with Amicon Ultra NMWCO 10 kDa centrifugal filters (Merck Millipore Ltd., Burlington, MA, USA) by centrifugation at 3200× *g* at 4 °C. Different samples, such as urine, cerebrospinal fluid, or plasma, required different times for concentration because they differed in chemical and physical properties, but for all of our plasma samples, the time was kept at a maximum of 45 min. The concentrated samples were then transferred to new tubes, and 1 mL was overlaid on the column followed by elution with filtered PBS, and twelve fractions of 500 µL were collected by gravity elution. Briefly, the isolation of EVs by SEC was performed in two rounds: first, it was determined in which fractions most EVs were eluted, and second, EV-enriched fractions were evaluated by protein quantification, nanoparticle tracking analysis, and transmission electron microscopy. The SEC method was applied to all samples for the insulation of EVs.

### 4.3. Nanoparticle Tracking Analysis (NTA)

To determine which SEC fractions were enriched in EVs, the particle concentration of individual fractions was analyzed by NTA, using NanoSight LM10 (Malvern Instrument Ltd., Malvern, Grovewood, UK) with specific parameters according to the manufacturer’s user manual (NanoSight LM10); this was performed for all samples belonging to the three groups. Samples were diluted at a ratio of 1:50 in filtered PBS to a final volume of 1 mL, and their concentration was adjusted by observing a particles/frame rate of around 50 (30–100 particles/frame). For each measurement, five consecutive 60 s videos were recorded under the following conditions: cell temperature −25 °C, syringe speed −22 µL/s (100 a.u.). Particles (EVs) were detected using a 488 nm laser (blue), and a scientific CMOS camera with an analysis threshold at 5. Among the pieces of information given by the software, the following were studied: mean size, mode (i.e., the most represented EV population size), and particles/mL.

### 4.4. Transmission Electron Microscopy (TEM)

Samples were processed as described previously [119], with a few changes. Briefly, 10 μL of vesicles were fixed in 2% paraformaldehyde (PFA) for 30 min and placed on formvar carbon-coated EM grids for 15 min. Then, samples were washed with H_2_O and fixed in 2.5% glutaraldehyde for 8 min, washed two times in H_2_O, and then stained with 2% uranyl acetate for 1.5 min. Samples were examined using a Jeol JEM1010 transmission electron microscope (Servicio Central de Soporte a la Investigación Experimental (SCSCIE), Universitat de València) at 80 kV and with a MegaView III digital camera. The images were recorded, and EV size was determined using the ImageJ software [120]. The TEM analysis was carried out on all EV samples and the statistical analysis was carried out considering only one enlargement for all the samples.

### 4.5. Western Blot Analysis

For Western blot analysis, the EV-enriched fractions were pooled and concentrated to a final volume of 250 µL with Amicon Ultra 0.5 mL centrifugal filters (Merck Millipore Ltd.) at 15,000× *g* for 1 h. Pellets were lysed using a radio-immunoprecipitation assay buffer (RIPA buffer, HEPES, NaCl, MGCl_2_, EDTA, Triton100, DTT, Na deoxycholate, SDS, NaF [80]) for 1 h on ice and then centrifuged for 20 min at 13,000× *g* at 4 °C. The protein concentration in samples was measured by the Bradford protein assay (BioRad, Hercules, CA, USA). Proteins were electro-transferred to nitrocellulose membranes (BioRad) with a transfer-blot semi-dry system (BioRad). Membranes were blocked with 5% bovine serum albumin (BSA, Sigma Aldrich, St. Louis, MO, USA) for 1 h. For EV detection by Western blot, we employed antibodies against CD81 tetraspanin (mouse anti-CD81, B-11 clone, Santa Cruz Biotechnology, Dallas, TX, USA; diluted 1:1000), TSG101 (mouse anti-TSG101, C2 clone, Santa Cruz Biotechnology; diluted 1:1000), albumin (mouse anti-albumin, F-10 clone, Santa Cruz Biotechnology; diluted 1:1000), cytochrome C (rabbit polyclonal anti-cytochrome C, H-105 clone, Santa Cruz Biotechnology; diluted 1:1000), Hsp70/Hsc70 (mouse anti-Hsp70/Hsc70, W27 clone, Santa Cruz Biotechnology; diluted at 1:500), and CT receptor (mouse monoclonal, 31/01-1H10-4-1-14 clone, BioRad, cat. N° MCA2191; diluted 1:500). The membranes obtained were detected using a Bio-Rad ChemiDoc MP imaging system (BioRad), the ImageJ software [121] was used for the analysis of band densities, and the values were expressed as arbitrary units (AU). Western blotting analysis was performed using three experimental replicates.

### 4.6. Immunohistochemistry

Immunohistochemical staining was performed on all 15 samples of GBM tissue and on 15 samples of normal tissue from the brain temporal lobe, using formalin-fixed paraffin-embedded blocks. Tissue sections, 5 µm thick, were obtained with a microtome, and then deparaffinized in xylene and hydrated by immersing in a series of graded ethanols (from ethanol 100% to 95%, 70%, 50%, and H_2_O). Antigen retrieval was performed by shaking the sections covered with epitope-retrieval solution (0.01 M citrate buffer, pH 6.0) for 8 min, and immersing them for 8 min in acetone at −20 °C to prevent the detachment of the sections from the slide. After washing the sections with PBS pH 7.4 for 5 min at 22 °C, the immunohistochemical reaction was performed via the streptavidin–biotin complex method using an Immunoperoxidase Secondary Detection System (Millipore, Burlington, MA, USA and Canada, cat. N DAB-500). The sections were then treated for 10 min with 3% hydrogen peroxide to inhibit endogenous peroxidase activity, and, after another wash with PBS at 22 °C for 5 min, they were treated by applying drops of blocking reagent (blue-colored reagent) for 5 min in an enclosed and humid container. Subsequently, endogenous peroxidase was inhibited by immersing the sections in 0.3% hydrogen peroxide for 10 min. Sections were then incubated with the primary antibody for CTR (mouse monoclonal, 31/01-1H10-4-1-14 clone, BioRad cat. N°: MCA2191; dilution 1:100). The following day, the sections were rinsed for 30 s with Rinse Buffer 1×, and incubated with a secondary antibody for 10 min at 22 °C. After washing again with Rinse Buffer 1×, the sections were incubated with streptavidin HRP for 10 min always in a humid and enclosed container. Subsequently, the slides were incubated in the dark for 10 min with an appropriate volume of chromogen reagent after another buffer rinse, and stained with Hematoxylin Counter Stain solution for 1 min at 22 °C for the nuclear blue counterstaining. Finally, the slides were mounted with coverslips using a permanent mounting medium (Vecta Mount, H-5000, Vector Laboratories, Inc., Burlingame, CA, USA). The slides were observed using an optical microscope (Microscope Axioscope 5/7 KMAT, Carl Zeiss, Milan, Italy) connected to a digital camera (Microscopy Camera Axiocam 208 color, Carl Zeiss, Milan, Italy) for evaluation of the immunopositivity, which appears in brown color. All observations were performed by two independent observers (F.C. and F.R.), who evaluated the immunostaining on two separate occasions and performed quantitative analysis to determine the percentage of immunopositivity. The percentage of positive cells was calculated in a high-power field (HPF) (magnification 400×) and repeated for 10 HPFs. The arithmetic mean ± standard deviation of counts was used for statistical analysis. The final percentage value for each case was the arithmetic mean of the 10 values obtained, and this arithmetic mean of counts was used for statistical analysis. Appropriate negative controls were run concurrently for each reaction. An IHC study was carried out on 10 sections of tumor tissue and 10 sections of healthy tissue.

### 4.7. Immunofluorescence and Confocal Microscopy

For the immunofluorescence experiments, primary and secondary cell lines [33] were used. Cells were placed in eight-well chamber slides, cultured for 24 h and fixed with ice-cold methanol for 30 min. The fixed cells were washed with PBS pH 7.4, and then incubated with unmasking solution (trisodium citrate 10 mM, 0.05% Tween-20, pH 6) for 10 min at 22 °C. After rinsing twice with PBS, cells were blocked with 3% (*w*/*v*) bovine serum albumin (BSA, Sigma Aldrich) in PBS for 30 min at 22 °C and incubated in a humidified chamber overnight at 4 °C with CT receptor primary antibodies (mouse monoclonal, 31/01-1H10-4-1-14 clone, BioRad cat. N° MCA2191; diluted 1:50). The day after, cells were washed twice in PBS, and were incubated with a fluorescent secondary antibody (mouse IgG antibody conjugated with FITC fluorescein isothiocyanate; Sigma-Aldrich; diluted 1:100). The nuclei were counterstained with DAPI 33342 (1:1000, Sigma-Aldrich) for 15 min at 22 °C. Finally, the slides were covered with drops of PBS and mounted with coverslips. The images were captured using a Leica Confocal Microscope TCS SP8 (Leica Microsystems, Tokyo, Japan).

### 4.8. LC-MS/MS Analysis

The peptide mixtures were analyzed for the spectral library acquisition by liquid chromatography (LC) using Ekspert nanoLC 425 (Eksigent Technologies, Dublin, CA, USA) connected to a mass spectrometer nanoESI qQTOF (6600 plus TripleTOF, ABSCIEX) in direct injection mode. Briefly, 7 µL of the peptide mixture sample was loaded on a trap column (LC Column, 12 nm, 3 µ Triart-C18, 0.5 × 5.0 mm; YMC) and desalted with 0.1% TFA at 10 µL/min for 5 min. Thereafter, the peptides were loaded onto an analytical column (LC Column, Luna Omega 3 µm Polar C18, 150 × 0.3 mm, Capillary Phenomenex) equilibrated in 3% acetonitrile 0.1% FA (formic acid). Then, peptide elution was carried out with a linear gradient of 3a35% B in A for 45 min (pooled samples) (A: 0.1% FA; B: ACN, 0.1% FA) at a flow rate of 5 μL/min. The eluted peptides were infused on a mass spectrometer nanoESI qQTOF (6600 plus TripleTOF, ABSCIEX). The samples were ionized using an Optiflow 1–50 μL Micro applying 4.5 kV to the spray emitter. The Survey MS1 was operated in data-dependent mode, in which a time of flight (TOF) mass spectrometry (MS) scan was carried out from 400 to 1250 *m*/*z* and accumulated for 200 ms. The quadrupole resolution was set to ‘UNIT’ for MS2 experiments, which were acquired from 100 to 1500 *m*/*z* for 20 ms in ‘high sensitivity’ mode. The criteria for precursor peptide ion selection were a charge of 2+ to 4+, and a minimum intensity of 300 counts per second (cps). Up to 75 ions were selected for fragmentation after each survey scan. Dynamic exclusion was set to 10 s. Ions with 1+ and unassigned charge states were removed from the analysis. The system sensitivity was controlled with 2 μg of HeLa trypsin digestion (Pierce, Appleton, WI, USA). Proteomic analysis was performed on all pathological and healthy samples.

### 4.9. Spectral Library Generation and Protein Quantitation

The .wiff data files obtained were processed using the ProteinPilot v5.0. search engine (Sciex). In ProteinPilot (version 5.0), ProteinPilot default parameters were used to generate a peak list directly from 6600plus TripleTof .wiff files. The Paragon algorithm [121] was used to search the NCBI (03.2018; 157,469,970 proteins searched) and SwissProt (06.2019; 1,120,570 proteins searched) with the following specific parameters: trypsin specificity, cys-alkylation, taxonomy restricted to human, and the search effort set to through. Irrespective of the peptide sequence assigned, and with the aim of avoiding using the same spectral evidence in more than one protein, the identified proteins were grouped based on tandem mass spectrometry (MS/MS) spectra by the Protein-Pilot Pro group algorithm. A protein group in a Pro Group Report is a set of proteins that share some physical evidence. Unlike sequence alignment analyses where full-length theoretical sequences are compared, the formation of protein groups in Pro Group is guided entirely by observed peptides only. Since the observed peptides are actually determined from experimentally acquired spectra, the grouping can be considered to be guided by the usage of spectra. Then, unobserved regions of the protein sequence play no role in explaining the data.

### 4.10. Statistical Analysis

Statistical analyses of the immunomorphological and biomolecular experiments were performed using one-way ANOVA with Bonferroni’s post hoc test (for all experiments) or Student’s *t*-test (only for immunohistochemistry, Figure 5D). All statistical analyses and graphs were performed using GraphPad Prism™ 4.0 software (GraphPad Software, San Diego, CA, USA). All data are presented as means ± SD of at least three independent experiments, with the level of statistical significance set at *p* ≤0.05.

## 5. Conclusions

Our data show that EVs can be isolated by SEC and demonstrate an enrichment in Hsp70 and CTR of the EVs isolated from GBM patients before surgery, in contrast to EVs from T1 and HVs samples, in which the two molecules were at very low levels or were undetectable (Figure 4A,B). We propose that the two proteins have potential as biomarkers for the differential diagnosis of tumor types and that their roles in carcinogenesis ought to be studied because, by elucidating their pro- and/or anti-tumor functions, avenues for developing specific treatment strategies will be opened. The use of EVs from liquid biopsies based on blood samples offers key advantages for screening patients with GBM in clinical practice, because it represents a non-invasive and rapid method that could have a more desirable impact on patient survival, with good quality of life. Furthermore, this scientific approach will contribute to the production of more robust and reliable preclinical data and thereby increase the therapeutic success rate for GBM of combined EVs-based clinical trials.

## Figures and Tables

**Table 1:** Characteristics of the patients and controls.

**15 Patients**	
**Age**	
Mean	66.5
Median (min–max)	61.0 (40.0–87.0)
Sex *	
Female	7 (46%)
Male	8 (54%)
**Tumor location in brain**	
Left frontal lobe	6 (41%)
Left parieto-occipital region	1 (6%)
Right frontal lobe	5 (33%)
IDH1 wild-type *	5 (33%)
**IDH1 mutant**	10 (66%)
Right temporal lobe	3 (20%)
**15 Controls Age (Years)**	
Mean	54.0
Median (min–max)	52.0 (49.0–68.0)
**Sex ^#^**	
Female	9 (60%)
Male	6 (40%)

* IDH1: isocitrate dehydrogenase (NADP(+)) 1. ^#^: The denominator used for calculating the percentages is 15 (n = 15).

## Data Availability

Data is contained within the article and supplementary material.

## Supplementary Materials

The following supporting information can be downloaded at: https://www.mdpi.com/article/10.3390/ijms25063415/s1.

## Abbreviations

CD, cluster of differentiation; CLR, calcitonin-like receptor; Cyt C, cytochrome C; CT, calcitonin; CTR, calcitonin receptor protein; EGFR, epidermal growth factor receptor; EVs, extracellular vesicles; GBM, glioblastoma; GBMT, glioblastoma tissue; GPCRs, G protein-coupled receptor; GSCs, glioma stem-like cells; Hsp, heat shock protein; HV, healthy volunteers; IDH, isocitrate dehydrogenase; IHC, immunohistochemistry; MHC, major histocompatibility complex; MVB, multivesicular bodies; NT, normal tissue; NTA, nanoparticle-tracking analysis; SEC, size-exclusion chromatography; Sp1, Sp1 transcription factor; TCL, total cell lysate; TEM, transmission electron microscope; TMZ, temozolomide; TP53, tumor protein P53; WHO, World Health Organization; CS, chaperone system.
